# Detection of Delafloxacin Resistance Mechanisms in Multidrug-Resistant *Klebsiella pneumoniae*

**DOI:** 10.3390/antibiotics14010062

**Published:** 2025-01-09

**Authors:** András Kubicskó, János Juhász, Katalin Kamotsay, Dora Szabo, Béla Kocsis

**Affiliations:** 1 Institute of Medical Microbiology, Semmelweis University, 1089 Budapest, Hungary; 2 Faculty of Information Technology and Bionics, Péter Pázmány Catholic University, 1083 Budapest, Hungary; 3 Central Microbiology Laboratory, National Institute of Hematology and Infectious Disease, Central Hospital of Southern-Pest, 1097 Budapest, Hungary; 4 HUN-REN-SU Human Microbiota Research Group, 1052 Budapest, Hungary; 5 Department of Neurosurgery and Neurointervention, Semmelweis University, 1085 Budapest, Hungary

**Keywords:** delafloxacin, fluoroquinolone resistance, high-risk clone, ESBL, NDM, WGS

## Abstract

**Background:** In this study, the mechanisms implicated in delafloxacin resistance in *Klebsiella pneumoniae* strains were investigated. Delafloxacin is a novel, broad-spectrum fluoroquinolone that has been approved for clinical application. **Methods:** In our study, 43 *K. pneumoniae* strains were assessed, antimicrobial susceptibility testing was performed via the broth microdilution method, and the minimum inhibitory concentration (MIC) values for ciprofloxacin, delafloxacin, levofloxacin, moxifloxacin, ceftazidime, cefotaxime, and imipenem were determined. Four delafloxacin-resistant *K. pneumoniae* strains were selected for whole-genome sequencing (WGS). **Results:** The MIC50 values for the 43 *K. pneumoniae* strains were as follows: ciprofloxacin 0.5 mg/L, levofloxacin 0.25 mg/L, moxifloxacin 0.5 mg/L, and delafloxacin 0.25 mg/L. All four selected delafloxacin-resistant *K. pneumoniae* strains showed extended-spectrum beta-lactamase production, and one strain exhibited carbapenem resistance. WGS enabled us to determine the sequence types (STs) of these strains, namely, ST307 (two strains), ST377, and ST147. Multiple mutations in quinolone-resistance-determining regions (QRDRs) were detected in all the delafloxacin-resistant *K. pneumoniae* strains; specifically, *gyrA* Ser83Ile and *parC* Ser80Ile were uniformly present in the strains of ST307 and ST147. However, in the ST377 strain, *gyrA* Ser83Tyr, Asp87Ala, and *parC* Ser80Ile, amino acid substitutions were detected. We also identified OqxAB and AcrAB efflux pumps in all delafloxacin-resistant *K. pneumoniae* strains. The association between beta-lactamase production and delafloxacin resistance was determined; specifically, CTX-M-15 production was detected in the ST147, ST307, and ST377 strains. Moreover, NDM-1 was detected in ST147. **Conclusions:** We conclude that multiple mutations in QRDRs, in combination with OqxAB and AcrAB efflux pumps, achieved delafloxacin resistance in *K. pneumoniae*. In our study, we report on NDM-1-producing *K. pneumoniae* ST147 in Hungary.

## 1. Introduction

*Klebsiella pneumoniae* is a member of the Enterobacteriaceae family; it is commonly found in the environment and can asymptomatically colonize the human nasopharynx and gastrointestinal tract [1]. However, *K. pneumoniae* is a major human pathogen, as it can cause a variety of diseases. Based on its pathogenic properties, *K. pneumoniae* is divided into classical and hypervirulent pathotypes. The classical *K. pneumoniae* pathotype is mainly acquired nosocomially among hospitalized patients and can cause bloodstream infections, ventilator-associated pneumonia, and catheter-associated urinary tract infections, among others. However, infections caused by hypervirulent *K. pneumoniae* show different clinical pictures, leading to severe community-acquired systemic and invasive infections and local infections (e.g., liver and extrahepatic abscesses) in otherwise healthy individuals [2,3,4,5,6,7,8].

Four major classes of virulence factors in *K. pneumoniae* have been characterized: capsule, including the production of a hypermucoviscosus capsule in hypervirulent *K. pneumoniae* strains; lipopolysaccharide (LPS); siderophores; and type 1 and 3 fimbriae [9]. The hypermucoviscous phenotype, in association with mucoviscosity-associated gene A (*magA*), is commonly seen in the K1, K2, K5, K20, K54, and K57 serotypes, with K1 and K2 being the most prevalent [5,10].

*K. pneumoniae* is included in the ESKAPEE pathogens (*Enterococcus faecium*, *Staphylococcus aureus*, *K. pneumoniae*, *Acinetobacter baumannii*, *Pseudomonas aeruginosa*, *Enterobacter* species, and *Escherichia coli*), which are capable of causing a large number of difficult-to-treat infections. In addition, these pathogens all exhibit multidrug resistance, which enables them to survive and spread in different environments, such as in hospitals [11,12,13]. The antibiotic resistance of *K. pneumoniae* is of great concern worldwide, as different resistance mechanisms can be acquired, and multidrug-resistant (MDR) *K. pneumoniae* clones can develop and spread across the globe. The World Health Organization (WHO) has grouped both carbapenem-resistant and third-generation cefalosporin-resistant *K. pneumoniae* as top-priority pathogens in a critical group, indicating the urgent need for research into new antibiotics and the development of effective treatments for infections caused by these pathogens [14]. Resistance to beta-lactams in *K*. *pneumoniae* is defined through the production of different beta-lactamases. One of the major classification systems used for beta-lactamases is the Ambler molecular classification, which separates the enzymes into four classes (A, B, C, and D) based on their amino acid sequences. Ambler classes A, C, and D include serine beta-lactamases, while class B consists of metallo-beta-lactamases (MBLs) [15]. In the class A group, the TEM, SHV, and CTX-M types are the prominent beta-lactamases. Currently, CTX-M-15 is the most widespread extended-spectrum beta-lactamase (ESBL) among clinical isolates. Ambler class A also includes serine carbapenemases, e.g., KPC and SME. Ambler class C includes AmpC-type beta-lactamases, e.g., CMY and DHA. Class D, or oxacillin-hydrolyzing (OXA) beta-lactamases, includes a broad spectrum of enzymes with different activity profiles; namely, several OXA-type beta-lactamases hydrolyze cefalosporins, but OXA-48 exhibits carbapenemase activity [15]. The Ambler class B enzymes are MBLs, which are different from the previously mentioned beta-lactamases because, at their active sites, there is at least one catalytically functional divalent zinc atom. MBLs are capable of hydrolyzing most beta-lactams (e.g., cefalosporins and carbapenems) but not monobactams. The most important MBLs are IMP-, VIM-, and NDM-type beta-lactamases [15]. The acquisition of ESBLs and MBLs in *K. pneumoniae* can occur through the uptake of plasmids. These resistance plasmids can carry different resistance genes that encode ESBLs (e.g., *bla*_TEM_, *bla*_SHV_, and *bla*_CTX-M-15_) and carbapenemases (e.g., *bla*_KPC_, *bla*_NDM_, and *bla*_VIM_) simultaneously; furthermore, other resistance genes that confer resistance to other antibiotics, such as aminoglycosides, fluoroquinolones, and colistin, can be present on these plasmids [1,16]. Resistance plasmids can be taken up and passed on to other bacteria because these plasmids can be easily transferred among the *Enterobacterales* species. The acquistion of resistance plasmids plays an important role in the development of MDR bacteria [17]. The treatment options for infections caused by multidrug-resistant *K. pneumoniae* are often limited to certain antibiotics. The use of newly approved antibiotic agents such as delafloxacin can help reduce the selective pressure that has been caused by the widespread use of beta-lactams [18]. Delafloxacin is one of the fluoroquinolone agents that has been approved for clinical application in recent years [19,20,21,22]. Delafloxacin exhibits promising in vitro activity against a broad range of bacteria, including Gram-positive, Gram-negative, and anaerobe bacteria [23,24,25]. Delafloxacin exhibits enhanced bactericidal efficacy against Gram-positive and Gram-negative bacteria because delafloxacin targets both bacterial DNA gyrase and topoisomerase IV enzymes with equal affinity. Delafloxacin has an improved active site compared to that of earlier fluoroquinolones, which allows it to act with greater potency on the binding sites of target molecules. Delafloxacin also inhibits the ability of *S. aureus* to form a biofilm [26]. Delafloxacin has also been approved for the treatment of several infections, including acute bacterial skin and skin structure infections (ABSSSIs) and community-acquired bacterial pneumonia (CABP); additional indications for delafloxacin therapy include complicated urinary tract infections and intra-abdominal infections [22,27].

Fluoroquinolone resistance mechanisms are classified into two main groups. The first one is mutation in the quinolone-resistance-determining regions (QRDRs) of bacterial chromosomes, namely, the coding genes of gyrase (*gyrA* and *gyrB*) and topoisomerase IV (*parC* and *parE*). Mutations in *gyrA* and *parC* tend to be more common than mutations of *gyrB* and *parE* [28]. AcrAB efflux pump has also been described as a chromosomally located fluoroquinolone resistance mechanism and is regulated by MarA, RamA, and SoxS determinants [29,30]. In addition to chromosomal mechanisms, plasmid-mediated quinolone resistance (PMQR) determinants have also been described; these confer reduced susceptibility to fluoroquinolones but enhance the development of chromosomal mutations, which lead to high-level fluoroquinolone resistance [31,32]. PMQRs include Qnr determinants, the aminoglycoside-acetyltransferase-(6′)-Ib-cr variant, and QepA and OqxAB efflux pumps. Qnr determinants are pentapeptide repeat proteins, which are capable of binding to gyrase and topoisomerase enzymes in order to protect them from fluoroquinolones. Qnr proteins are clustered into several groups based on their amino acid homology and include QnrA, QnrB, QnrC, QnrD, and QnrE. The aminoglycoside-acetyltransferase-(6′)-Ib-cr (*aac(6′)-Ib-cr*) variant leads to the enzymatic modification of certain fluoroquinolone agents (e.g., ciprofloxacin and norfloxacin); this reduces the antibacterial efficacy of these agents. QepA and OqxAB efflux pumps extrude fluoroquinolones, together with other bactericidal agents, from the bacterial cell [33,34,35,36,37,38].

This study aimed to investigate the antibacterial efficacy of delafloxacin against *K. pneumoniae* and analyze the mechanisms that confer resistance to delafloxacin in *K. pneumoniae* strains.

## 2. Results

### 2.1. Antimicrobial Susceptibility Testing

**Figure 1:**
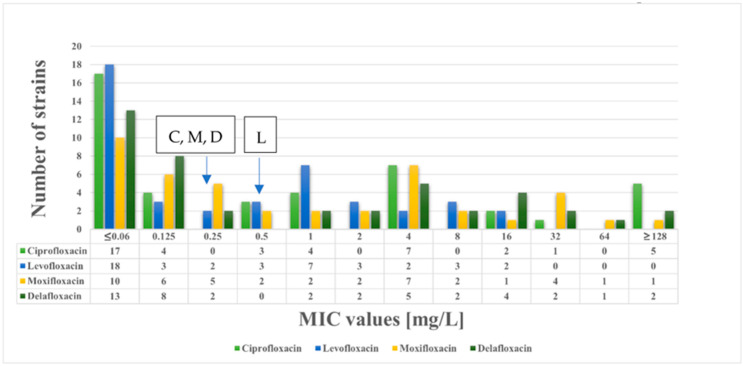
Distribution of MIC values for ciprofloxacin, levofloxacin, moxifloxacin, and delafloxacin among the 43 *K. pneumoniae* strains. Breakpoints are shown for ciprofloxacin (C), levofloxacin (L), moxifloxacin (M), and delafloxacin (D). EUCAST breakpoints were used for ciprofloxacin, levofloxacin, and moxifloxacin. In the case of delafloxacin, the FDA breakpoint was applied.

**Figure 2:**
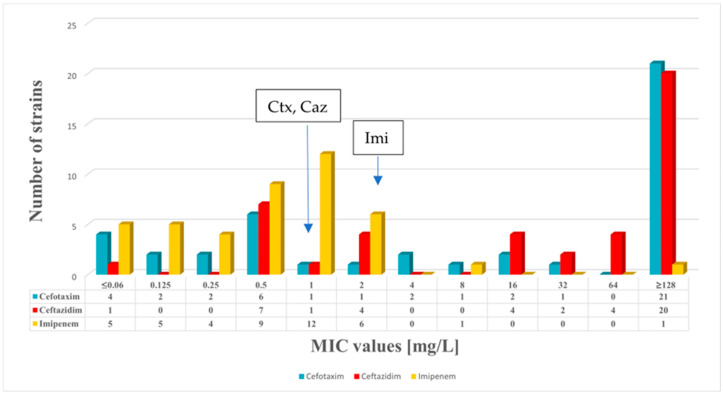
Distribution of MIC values for cefotaxime, ceftazidime, and imipenem among the 43 *K. pneumoniae* strains. Breakpoints are shown for cefotaxime (Ctx), ceftazidime (Caz), and imipenem (Imi). EUCAST breakpoints were used for cefotaxime, ceftazidime, and imipenem.

In total, 43 *K. pneumoniae* clinical isolates were included in our study. In addition, the minimum inhibitory concentration (MIC) values of fluoroquinolones and beta-lactams were determined. The distributions of the fluoroquinolone and beta-lactam MIC values are shown in Figure 1 and Figure 2, respectively. Among the strains included in this study, 21 were susceptible to ciprofloxacin, 26 were susceptible to levofloxacin, 21 were susceptible to moxifloxacin, and 23 were susceptible to delafloxacin. Altogether, 20 *K. pneumoniae* strains exhibited resistance to delafloxacin, and these strains were also resistant to the other fluoroquinolones tested.

In this collection of *K. pneumoniae* strains, an association between fluoroquinolone resistance and ESBL positivity was detected. Altogether, 47% of the *K. pneumoniae* strains were fluoroquinolone-resistant and showed an ESBL phenotype in this study. In this collection of strains, 16% of *K. pneumoniae* exhibited an ESBL phenotype but remained susceptible to fluoroquinolone. Two *K. pneumoniae* strains were resistant to imipenem in this study.

The MIC50 values of the 43 *K. pneumoniae* strains were as follows: ciprofloxacin 0.5 mg/L, levofloxacin 0.25 mg/L, moxifloxacin 0.5 mg/L, and delafloxacin 0.25 mg/L.

The MIC values were interpreted based on the EUCAST recommendations for ciprofloxacin, levofloxacin, and moxifloxacin. The MIC values of delafloxacin were interpreted based on the FDA recommendation [22].

### 2.2. Genome Sequencing

The whole-genome sequencing (WGS) analysis of four delafloxacin-resistant *K. pneumoniae* strains found that these strains belong to different sequence types (STs) according to the multi-locus sequence typing (MLST); these sequence types were ST307 (two strains), ST377, and ST147. Different beta-lactamase genes were detected in these strains; in ST307, these genes were *bla*_SHV-28_, *bla*_OXA-1_, *bla*_TEM-1B_, and *bla*_CTX-M-15_; in ST377, these genes were *bla*_OXA-1_, *bla*_SHV-110_, and *bla*_CTX-M-15_; and, in ST147, these genes were *bla*_SHV-11_, *bla*_OXA-1_, *bla*_OXA-9_, *bla*_TEM-1A_, *bla*_CTX-M-15_, and *bla*_NDM-1_ (Table 1).

The WGS analysis detected multiple QRDR mutations and PMQR determinants in delafloxacin-resistant *K. pneumoniae* strains. The following QRDR alteration was detected in the ST147 and ST307 strains: *gyrA* Ser83Ile. In contrast, double *gyrA* mutations were detected in ST377, namely, Ser83Tyr and Asp87Ala. In the case of the *parC* mutation, Ser80Ile was uniformly present in all delafloxacin-resistant *K. pneumoniae* strains of ST147, ST307, and ST377. Additionally, other mutations in ParC that may play a role in delafloxacin resistance were detected; specifically, a Pro402Ala amino acid substitution was present in all four delafloxacin-resistant *K. pneumoniae* strains. Another amino acid substitution in ParC Asn304Ser was present in the ST307 and ST147 strains.

Different PMQR determinants were detected in delafloxacin-resistant *K. pneumoniae* strains. The following determinants were detected: *qnrS1* in the ST147, *qnrB1* and *aac(6′)-Ib-cr* in both strains of ST307*,* and *aac(6′)-Ib-cr* in the ST377 strain. All tested *K. pneumoniae* strains had OqxAB and AcrAb/TolC efflux pumps (Table 2). According to an analysis of the plasmid-coded resistance genes of delafloxacin-resistant *K. pneumoniae* strains, the following resistance determinants were located on plasmids: *qnrS1*, *qnrB1, aac(6′)-Ib-cr, bla*_OXA-1_, *bla*_OXA-9_, *bla*_TEM-1A,_ *bla*_TEM-1B,_ *bla*_CTX-M-15_, and *bla*_NDM-1_. The WGS analysis detected virulence determinants in delafloxacin-resistant *K. pneumoniae* strains. These virulence determinants are shown in Table 3**.**

**Figure 3:**
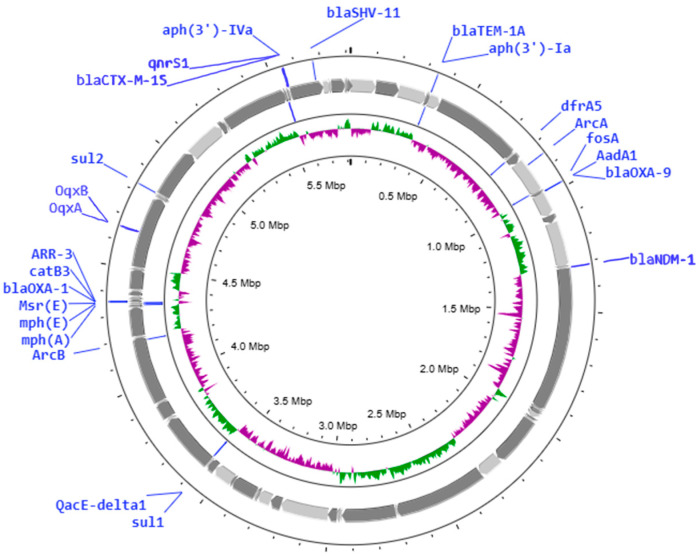
Genome characteristics of the NDM-1-producing *K. pneumoniae* ST147 strain. Major resistance genes are indicated in the figure. The circos plot shows the GC content skew in the inner circle histograms, the contigs (with grey arrows), and the positions of the detected resistance genes.

**Figure 4:**
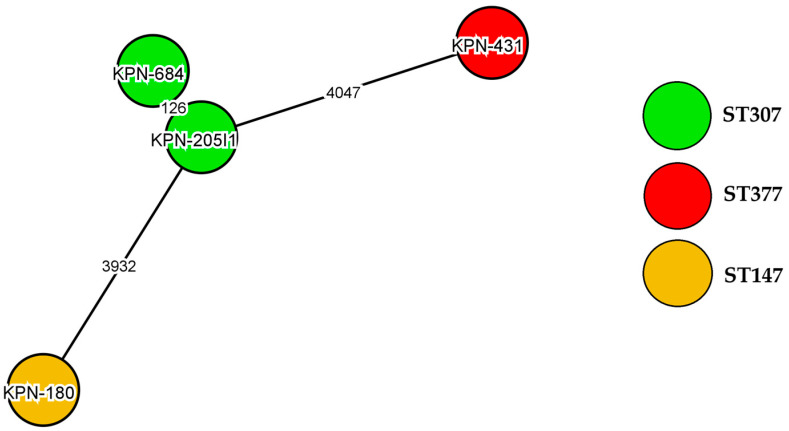
The wgMLST of four delafloxacin-resistant *K. pneumoniae* strains. Each circle indicates a *K. pneumoniae* strain. Lines between the circles denote differences in the allelic variant between the clones. Each clone is shown in a different color.

The genome structure of the NDM-1- and CTX-M-15-producing delafloxacin-resistant *K. pneumoniae* ST147 strain is shown in Figure 3. The whole-genome multi-locus sequence typing (wgMLST) data of four delafloxacin-resistant *K. pneumoniae* strains are analyzed in Figure 4.

## 3. Discussion

Antibiotic resistance poses a significant challenge worldwide; according to current estimations, 4.95 million patients die yearly due to antibiotic-resistant bacterial infections [39]. Multidrug-resistant *K. pneumoniae* is one of the major pathogens responsible for nosocomial infections; this is because it can cause various difficult-to-treat infections and a limited number of effective antibiotics are available for treatment. It is clear in clinical departments worldwide that new, effective antibiotics are needed. Therefore, in order to overcome the challenge of antibiotic resistance, new antibiotics are being introduced to treat such infections. Agents from different antibiotic groups are among the new antibiotics that have been approved for clinical use in recent years. Notably, beta-lactam and beta-lactamase inhibitor combinations (e.g., ceftazidime–avibactam, ceftolozane–tazobactam, and meropenem–vaborbactam), plazomicin from the aminoglycoside group, and new fluoroquinolones have also been approved for clinical application [40].

Delafloxacin is a new broad-spectrum fluoroquinolone agent that has a larger molecular surface compared to earlier fluoroquinolones, which enhances its potency. Delafloxacin has been approved for clinical application; however, the resistance of *K. pneumoniae* to delafloxacin has not yet been thoroughly investigated. In earlier studies, delafloxacin resistance was analyzed in *Neisseria gonorrhoeae*, *S. aureus*, and *Helicobacter pylori* [24,41,42,43]. In addition, we previously investigated the mechanisms implicated in delafloxacin resistance in high-risk clones of *E. coli.* In these earlier investigations, multiple QRDR mutations associated with PMQR determinants were detected in delafloxacin-resistant *E. coli* strains [44,45].

In our current study, we analyzed the resistance mechanisms of delafloxacin-resistant *K. pneumoniae* clinical isolates. Altogether, 43 *K. pneumoniae* isolates were included in this study and the distribution of the MIC values of fluoroquinolone was determined. The MIC50 values were 0.25 mg/L for delafloxacin, which is lower than that of ciprofloxacin and moxifloxacin (0.5 mg/L) but equal to levofloxacin (0.25 mg/L). However, considering that delafloxacin has a unique chemical structure compared to earlier fluoroquinolones, based on the MIC50 values, it can be assumed that delafloxacin has more potent effects on *K. pneumoniae* than ciprofloxacin or moxifloxacin.

Four delafloxacin-resistant *K. pneumoniae* strains were investigated via WGS in order to analyze their molecular mechanisms and genetic markers. Three out of the four strains belonged to internationally disseminated high-risk clones, namely, ST147 (one strain) and ST307 (two strains). However, one strain represented ST377, which has previously been reported to be a multidrug-resistant clone in certain countries [46,47,48].

*K. pneumoniae* ST147 clones are well-known international high-risk pathogens with MDR phenotypes that are associated with several carbapenemases; in some cases, the co-production of KPC-2, NDM-1, and OXA-48 has been established [49]. ST147 first appeared in Hungary in 2008 [50]; then, in the following years, it appeared in Italy, Greece, India, and North African countries [51,52]. ST147 strains were resistant to fluoroquinolones, with *gyrA* S83I and *parC* S80I QRDR mutations being associated with beta-lactamases such as VIMs, KPC-2, NDM-1, and OXAs. Nosocomial outbreaks of this clone were reported in several countries due to the appearance of acquired antimicrobial resistance determinants; these included SHV-36, CTX-M-15, KPCs, NDMs, VIMs, OXA-48, OXA-181, and OXA-204 [53,54].

*K. pneumoniae* ST307 is also an internationally disseminated high-risk clone. ST307 was reported in Hungary in 2019, with this strain found to be positive for CTX-M-15 and resistant to fluoroquinolone [55]. However, ST307 has disseminated worldwide, and it has been reported in Italy, Columbia, the United States of America, and South Africa [52]. ST307 has been found to exhibit different resistance mechanisms; in recent years, carbapenemases-producing strains of ST307 have been reported, namely, production of NDM-1, OXA-181, OXA-48, and KPC-2 has been detected [56,57,58,59,60].

In our study, an association between beta-lactamase genes and delafloxacin resistance was detected in all four *K. pneumoniae* strains via WGS. ST147 possessed *bla*_NDM-1_, *bla*_SHV-11_, *bla*_OXA-1_, *bla*_OXA-9_, *bla*_TEM-1A_, and *bla*_CTX-M-15_; both strains of ST307 possessed *bla*_SHV-28_, *bla*_OXA-1_, *bla*_TEM-1B_, and *bla*_CTX-M-15_; and ST377 possessed *bla*_OXA-1_, *bla*_SHV-110_, and *bla*_CTX-M-15_.

This study aimed to detect and analyze the genetic markers of delafloxacin resistance in *K. pneumoniae* strains. Among the fluoroquinolone resistance markers detected, all strains of internationally disseminated high-risk clones, namely, ST147 and ST307, uniformly presented *gyrA* Ser83Ile and *parC* Ser80Ile. However, ST377 possessed *gyrA* Ser83Tyr, Ser87Ala, and *parC* Ser80Ile. All delafloxacin-resistant *K. pneumoniae* strains carried *oqxAB* and *acrAB* efflux pumps.

**Figure 5:**
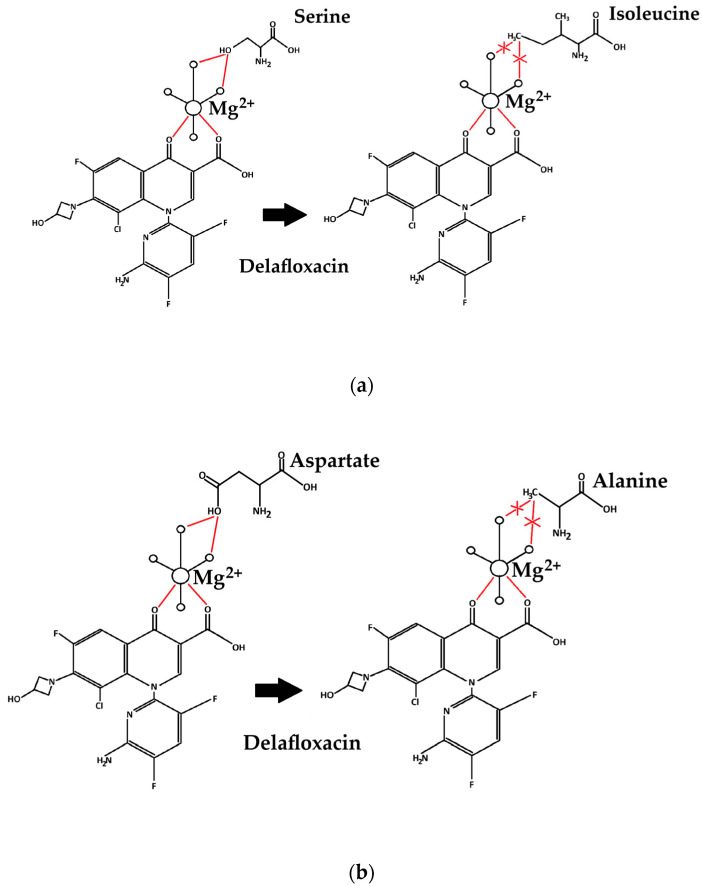

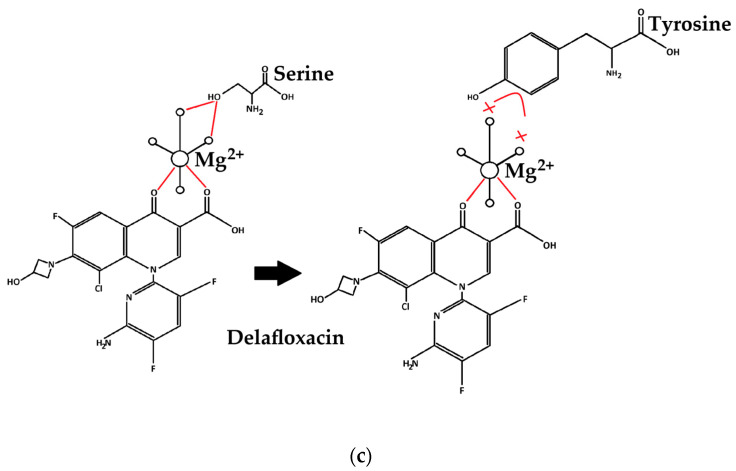
Role of amino acid substitutions in gyrase and topoisomerase enzymes in correlation with the action of delafloxacin. This figure illustrates the substitution of serine to isoleucine (**a**), the substitution of aspartate to alanine (**b**), and the substitution of serine to tyrosine (**c**) in correlation with the action of delafloxacin.

An analysis of the chemical structure of the detected QRDR mutations revealed key insights into the development of a lower target-binding affinity of delafloxacin, which would ultimately lead to resistance. At position 83 in GyrA, a polar serine amino acid changes to an aromatic tyrosine molecule or to isoleucine; this indicates substitution to a hydrophobic amino acid. At position 87 in GyrA, the polar amino acid aspartate is substituted to alanine, which is a hydrophobic molecule. In ParC, the substitution of serine (polar) at position 80 to isoleucine (hydrophobic) increases the MIC values of delafloxacin (Figure 5).

Earlier studies found that a dissimilar fitness cost is observed among different clones of *K. pneumoniae* during the development of fluoroquinolone resistance [61]. Generally, the development of QRDR mutations results in an energy burden on strains of *K. pneumoniae*. However, high-risk internationally disseminated clones retain their fitness, which enables them to survive and disseminate. Specifically, double serine mutations in QRDRs were found to be a possible marker of major internationally disseminated clones that retain their fitness [62]. Interestingly, in our study, all delafloxacin-resistant *K. pneumoniae* strains harbored a double-serine mutation in QRDRs, which indicates that delafloxacin resistance can occur in major internationally disseminated *K. pneumoniae* clones.

## 4. Materials and Methods

### 4.1. Strains

In this study, 43 nonrepetitive *K. pneumoniae* strains were collected between September and December 2022 at South-Pest Central Hospital, National Institute of Hematology and Infectious Diseases, Budapest, Hungary. These *K. pneumoniae* strains were isolated from different clinical specimens, including blood culture and urine samples. All specimens were processed as part of routine laboratory procedures and the strains were selected based on inclusion criteria. The identification of isolates was performed using matrix-assisted laser desorption ionization time-of-flight mass spectrometry (MALDI Biotyper, Bruker, Bremen, Germany). In this study, *K. pneumoniae* strains were included if they exhibited resistance to ciprofloxacin and/or resistance to third-generation cephalosporins or if their ESBL positivity had been confirmed via a double-disk synergy test.

### 4.2. Detection of the Minimum Inhibitory Concentration (MIC)

All *K. pneumoniae* strains were investigated via the broth microdilution method in order to determine the MIC values for delafloxacin, ciprofloxacin, levofloxacin, moxifloxacin, ceftazidime, cefotaxime, and imipenem. We performed antibiotic susceptibility testing using Muller–Hinton broth in 96-well microplates. The MIC results for ciprofloxacin, levofloxacin, moxifloxacin, ceftazidime, cefotaxime, and imipenem were interpreted according to the latest EUCAST protocol v14.0 (www.eucast.org (accessed on 1 January 2024)). However, the MIC values for delafloxacin were interpreted based on U.S. Food and Drug Administration (FDA) recommendations [22]. We used *E. coli* ATCC 25922 as a control strain in this study (https://www.atcc.org/products/25922).

### 4.3. Whole-Genome Sequencing (WGS)

In this study, WGS analysis was performed on four selected *K. pneumoniae* strains to detect resistance determinants. The selection criteria for WGS on *K. pneumoniae* strains were as follows: resistance to ciprofloxacin and delafloxacin, an ESBL phenotype, and the resistance of one *K. pneumoniae* strain to imipenem. The Illumina MiSeq platform was used to perform WGS at Eurofins BIOMI Kft (Gödöllő, Hungary). The genomic DNA of all four *K. pneumoniae* strains was extracted using the NucleoSpin Microbial DNA Mini kit (Macherey-Nagel, Düren, Germany). A qubit fluorometer was used to measure the isolated bacterial DNA, and then the quality of the DNA was checked via microcapillary electrophoresis (Tape Station 4150, Agilent, Waldbronn, Germany). The Illumina DNA Prep kit (San Diego, CA, USA) was used to prepare libraries. The Illumina Miseq system was used for sequencing, and the MiSeq Reagent Kit v2 was employed to generate 250 bp paired-end reads. SPAdes Genome assembler algorithm v3.15.3 was used for the genome assembly of *K. pneumoniae* strains. Antibiotic-resistant genes were detected in the assembled genomes using Bionumerics v8.1 software [44,45]. We also used the Comprehensive Antibiotic Resistance Database (CARD) and Resistance Gene Identifier program for the detection of resistance genes at https://card.mcmaster.ca/analyze/rgi (accessed on 10 September 2024).

### 4.4. Analysis of Genome Sequence

The assembled genomes of *K. pneumoniae* strains were analyzed using traditional seven-gene multi-locus sequence typing (MLST) [63]. Whole-genome MLST (wgMLST) was performed via Bionumerics v8.1 based on accessory schema with 19086 loci; this was complemented with the 634 core loci [64], 7 MLST loci [41], and 2 capsular typing loci (*wzc* [65] and *wzi* [66] sequencing) allele variants.

The genome characteristics of *K. pneumoniae* ST147 are shown in Figure 3. The plot was created using the Proksee server (https://proksee.ca/) (accessed on 10 September 2024) [67] based on the genome annotation generated by RAST (https://rast.nmpdr.org/rast.cgi) (accessed on 10 September 2024) [68,69] for *K. pneumoniae*, with default parameters.

## 5. Conclusions

Our study demonstrates that multiple mutations in QRDRs are present in delafloxacin-resistant *K. pneumoniae*, namely, *gyrA* Ser83Ile, *parC* Ser80Ile or *gyrA* Ser83Tyr, Ser87Ala, and *parC* Ser80Ile. It can be assumed that multiple specific amino acid substitutions mediate delafloxacin resistance; notably, at position 83 in GyrA, a polar serine is substituted with an apolar isoleucine or tyrosine; at position 87 in GyrA, an aspartate is substituted with alanine; and, at position 80 in ParC, a polar serine is substituted with an apolar isoleucine. We conclude that both *gyrA* and *parC* mutations in combination with OqxAB and AcrAB efflux pumps are needed to achieve delafloxacin resistance in *K. pneumoniae*. In our study, we report the detection of an NDM-1-producing *K. pneumoniae* ST147 strain in Hungary.

## Figures and Tables

**Table 1:** Genetic determinants of four delafloxacin-resistant *K. pneumoniae* strains. MLST: multi-locus sequence typing; PMQR: plasmid-mediated quinolone resistance determinants; beta-lactamases; MIC values of ciprofloxacin (Cip); levofloxacin (Lev); moxifloxacin (Mox); delafloxacin (Del); ceftazidime (Caz); cefotaxime (Ctx); and imipenem (Imi). All MIC values are shown in mg/L.

	MLST	PMQR	Beta-Lactamases	Cip	Lev	Mox	Del	Caz	Ctx	Imi
***K. pneumoniae* 180**	ST147	*qnrS1,* *oqxA, oqxB*	*bla*_NDM-1_, *bla*_SHV-11_, *bla*_OXA-1_, *bla*_OXA-9_, *bla*_TEM-1A_, *bla*_CTX-M-15_	128	16	128	128	128	128	128
***K. pneumoniae* 205/1**	ST307	*qnrB1,* *aac(6* *′)-Ib-cr, * *oqxA, oqxB*	*bla*_SHV-28_, *bla*_OXA-1_, *bla*_TEM-1B_, *bla*_CTX-M-15_	128	8	32	64	128	128	1
***K. pneumoniae* 431**	ST377	*aac(6* *′)-Ib-cr, * *oqxA, oqxB*	*bla*_SHV-110_, *bla*_OXA-1_, *bla*_CTX-M-15_	128	8	32	4	128	128	1
***K. pneumoniae* 684**	ST307	*qnrB1,* *aac(6* *′)-Ib-cr, * *oqxA, oqxB*	*bla*_SHV-28_, *bla*_OXA-1_, *bla*_TEM-1B_, *bla*_CTX-M-15_	128	8	32	32	128	128	0.5

**Table 2:** Four delafloxacin-resistant *K. pneumoniae* strains with fluoroquinolone resistance determinants. PMQR: plasmid-mediated quinolone resistance determinants, QRDR: quinolone-resistance-determining region, ST: sequence type.

ST	ST377	ST307	ST307	ST147
**PMQR**	*aac(6′)-Ib-cr*, *oqxA, oqxB*	*qnrB1*, *aac(6′)-Ib-cr*, *oqxA, oqxB*	*qnrB1*, *aac(6′)-Ib-cr, oqxA, oqxB*	*qnrS1*, *oqxA, oqxB*
**QRDR**	*gyrA*: Ser83Tyr, Asp87Ala *parC*: Ser80Ile, Pro402Ala	*gyrA*: Ser83Ile *parC*: Ser80Ile, Asn304Ser, Pro402Ala	*gyrA*: Ser83Ile *parC*: Ser80Ile, Asn304Ser, Pro402Ala	*gyrA*: Ser83Ile *parC*: Ser80Ile, Asn304Ser, Pro402Ala
**Efflux system**	AcrAB/TolC	AcrAB/TolC	AcrAB/TolC	AcrAB/TolC
**delafloxacin MIC (mg/L)**	4	32	64	128

**Table 3:** Virulence determinants of four delafloxacin-resistant *K. pneumoniae* strains.

ST147	ST307	ST307	ST377
**Siderophores** *fyu, iucB, ybtA,* *ybtE, ybtP, ybtQ, ybtS, ybtT, ybtU, ybtX*			**Siderophores** *fyuA, irp1, irp2, ybtA, ybtE, ybtP, ybtQ, ybtS, ybtT, ybtU, ybtX*
**Type III fimbriae** *mrkA, mrkC, mrkD, mrkF, mrkH, mrkI, mrkJ*	**Type III fimbriae** *mrkA, mrkC, mrkD, mrkF, mrkH, mrkI, mrkJ*	**Type III fimbriae** *mrkA, mrkC, mrkD, mrkF, mrkH, mrkI, mrkJ*	**Type III fimbriae** *mrkA, mrkC, mrkD, mrkF, mrkH, mrkI*
**Mucus regulator** *rmpA* *rmpA2*			

## Data Availability

Genomic data of four delafloxacin-resistant *K. pneumoniae* are submitted to NCBI Genbank at the following BioProject number: PRJNA1189473, and at the following accession numbers SRA: SAMN4497125 (Kpn 180); SAMN44971254 (Kpn 250/1); SAMN44971255 (Kpn 431); SAMN44971256 (Kpn 684).
